# Therapy-Induced Growth and Sexual Maturation in a Developmentally Infantile Adult Patient with a PROP1 Mutation

**DOI:** 10.3389/fendo.2017.00309

**Published:** 2017-11-13

**Authors:** Ludmila Brunerova, Ivana Cermakova, Bozena Kalvachova, Jana Skrenkova, Renata Poncova, Petr Sedlak

**Affiliations:** ^1^Faculty Hospital Královské Vinohrady, 3rd Faculty of Medicine, II. Department of Internal Medicine, Prague, Czechia; ^2^Institute of Endocrinology, Prague, Czechia; ^3^1st Faculty of Medicine, Department of Obstetrics and Gynecology, General Teaching Hospital, Prague, Czechia; ^4^Faculty of Science, Department of Anthropology and Human Genetics, Charles University, Prague, Czechia

**Keywords:** growth, sexual maturation, PROP1 mutation, hormonal substitution therapy, growth hormone therapy

## Abstract

**Background:**

Hypopituitarism as a result of PROP1 (prophet of PIT1) mutation represents the most common genetic cause of combined deficiency of pituitary hormones and due to growth retardation it is typically diagnosed in childhood.

**Case description:**

We present a unique case report of a prepubertal woman with growth retardation in whom combined pituitary hormone deficiency [central hypopituitarism, hypogonadism, and growth hormone (GH) deficiency] caused by homozygous mutation c.150delA in the PROP1 gene was diagnosed late in young adulthood due to unfavorable life circumstances. Through cautiously combined GH therapy and sex hormone therapy, she has achieved better than expected height (exceeding predictions based on family height) and sexual maturation, including regular menstrual cycles.

**Conclusion:**

Early diagnosis of panhypopituitarism due to PROP1 mutation is essential for successful treatment; however, our case report shows that carefully titrated GH treatment and sex hormone substitution, although initiated in adulthood, enable restoration of physiological growth and sexual development in a hormonally infantile adult woman with a PROP1 mutation.

## Introduction

Combined pituitary hormone deficiency (CPHD) is defined as missing production of two or more pituitary hormones and is sporadic in majority of cases; however, familial forms (with different manners of inheritance) have been also described (1). Although several genes which are involved in pituitary cell differentiation (e.g., PIT-1, PROP1, LHX-3, LHX-4, HESX-1, SIX-6, OTX-2, PTX-2, GLI-2, and SOX-3) were identified as genetic causes of CPHD, their overall frequency among the cases is low, with mutation of PROP1 being the most common one (2, 3).

PROP1 is the abbreviation from English “prophet“ (precursor) of the PIT1 transcription factor responsible for development of the anterior part of the pituitary lobe during embryogenesis. PIT1 is a homeobox protein encoded by the PROP1 gene, essential for the proper maturation of specialized cell types in the anterior part of the pituitary gland which produce and secrete polypeptide hormones in response to stimulation signals from the hypothalamus and stimulatory feedback from target organs (4).

Mutation of PROP1 leads to CPHD of all pituitary hormones: growth hormone (GH), thyroid-stimulating hormone (TSH), luteinizing hormone (LH), follicle-stimulating hormone (FSH), prolactin (PRL), and adrenocorticotropic hormone (ACTH) (1) and represents probably the most common genetic cause affecting 18–24% of tested patients with CPHD; in familial forms inherited in an autosomal recessive manner (1–3).

Clinical and hormonal phenotype is highly variable, but typically, PROP1 gene mutation is manifested as hypopituitarism with growth failure as the first sign and thus it is detected in early childhood. Central hypothyroidism usually appears later in childhood and varies in its severity. Delayed or absent sexual maturation is observed in the second decade of life. ACTH insufficiency is less common and, if it occurs, it is more likely in adulthood (5–7). In general, no correlation has been observed between different PROP1 pathogenic variants and the phenotype (6–8).

Diagnosis of CPHD is based on the detection of hormonal deficiencies using standard basal hormonal examinations and challenge tests. Mutation of PROP1 can then be confirmed by molecular-genetic testing (6, 9) using sequence analysis, which detects different gene variants classified with respect to their phenotypic impact as benign, likely benign, of uncertain significance, likely pathogenic or pathogenic, or by other methods [e.g., quantitative polymerase chain reaction (PCR) or chromosomal microarray].

Magnetic resonance imaging (MRI) is the technique of choice in the diagnosis of patients with CPHD. Although the correlation of genetic mutations with endocrine and MRI phenotypes has improved the management of some patients with CPHD (10), pituitary morphology, particularly in PROP1 mutation, is not very specific and varies widely from a small to an enlarged gland (11).

There is no causal treatment for PROP1 mutation cases; the therapy is focused on substitution of all the lacking hormones while respecting developmental aspects (6, 8, 12).

We present a case of an adult woman with delayed diagnosis of CPHD secondary to PROP1 mutation, who due to certain life circumstances was still prepubertal but responded well to hormonal treatment. To the best of our knowledge, our case is exceptional in terms of growth as a result of GH treatment initiated in adulthood.

## Case Presentation

The patient signed an informed consent statement and agreed on the examination as well as the presentation of her case, including the picture.

The patient was a young woman born in Ukraine in 1985. She was her mother’s second uncomplicated pregnancy, with a term physiological delivery (3,900 g, 53 cm). Her parents and two sisters were healthy and of normal height (mother 158 cm, father 165 cm, and sisters both 170 cm). Unfortunately, no medical records from Ukraine are available; the only source of information about her health status is the self-report by our patient. She stated having been treated with GH from 15 to 17 years due to growth retardation and gained 25 cm of height. At the same time, she was treated with iodine (Jodomarin) 100 µg daily. No other hormonal treatment was indicated in Ukraine at that time. She moved to Czechia at the age of 25 years.

**Figure 1:**
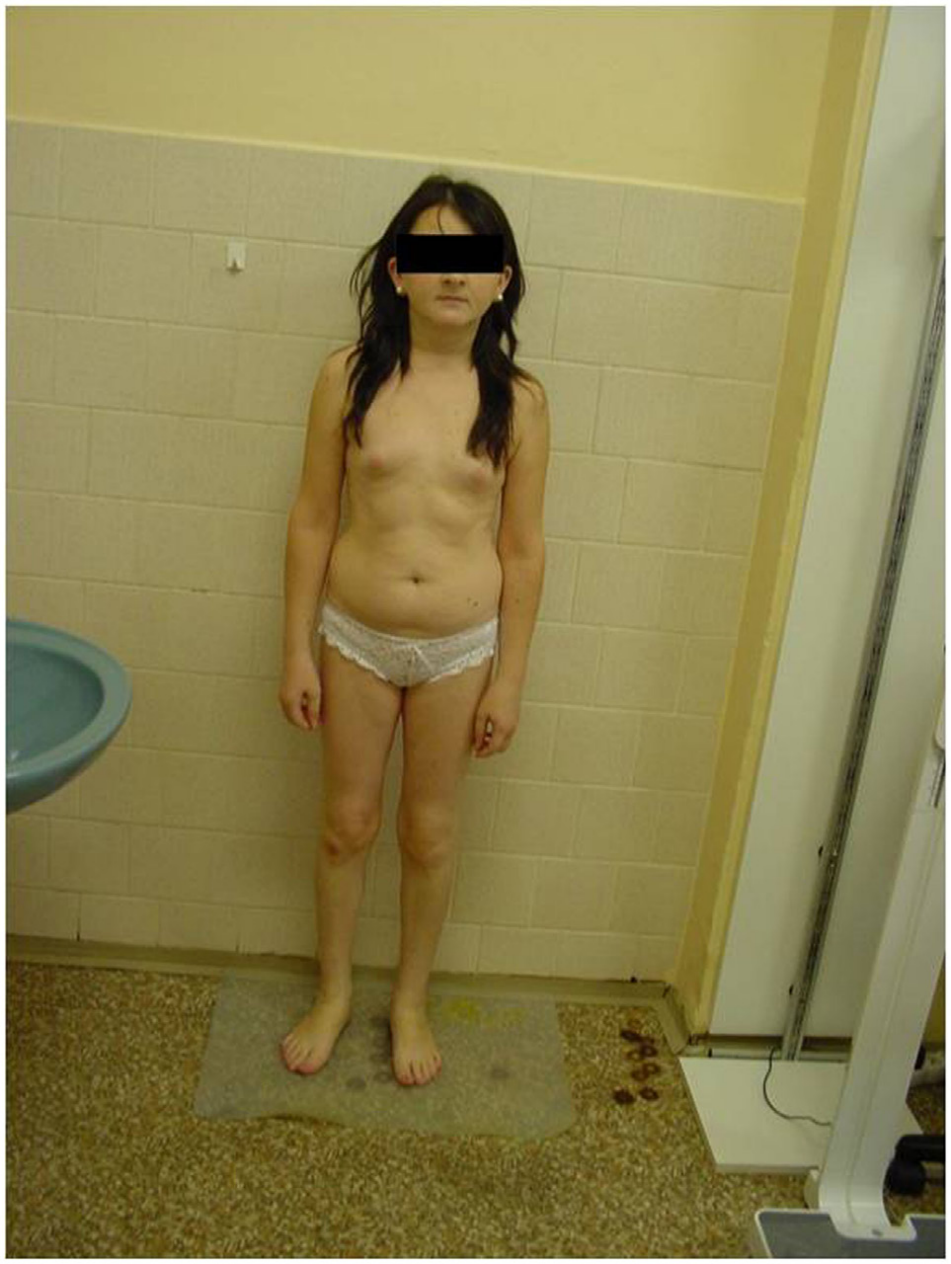
Picture of our patient at the age of 27 years.

She was referred by her GP to an endocrinologist for suspected hypopituitarism (small stature and primary amenorrhea) in May 2013. Surprisingly, the patient herself did not express any concerns either about her childish appearance or the absence of menstrual periods, or about the lack of secondary sexual signs including pubic and axillary hair and breasts. She denied perceiving any potential symptoms connected with hypothyroidism or hypocortisolism. However, during examination she displayed an infantile appearance (149 cm, 43 kg), pale skin, puffy face, no axillary or pubic hair (Tanner AH1 and PH1, respectively), no breast development (Tanner B1-2), and with no history of a menstrual cycle (Figure 1).

### Laboratory Assessment

#### Methods

The laboratory assessment was performed in the Laboratory of the Institute of Endocrinology using the following methods presented in the form: hormone, method, producer, and reference range––in relevant hormones corrected for age and gender and menstrual cycle phase: fT3 [electroluminescence (ECLIA), Roche, 3.1–6.8 pmol/L]; fT4 (ECLIA, Roche, 12–22 pmol/L); TSH (ECLIA, Roche, 0.27–4.2 mIU/L); estradiol (ECLIA, Roche, reference range: 0.0454–0.854 nmol/L follicular phase); FSH (ECLIA, Roche, 3.5–12.5 IU/L follicular phase); LH (ECLIA, Roche, 2.4–12.6 IU/L follicular phase); S-cortisol (RIA, Immunotech, 263–724 nmol/L); ACTH (ECLIA, Roche, 7.2–63.3 ng/L); androstendione [radioimmunoassay (RIA), Immunotech, 2.47–9.4 nmol/L]; DHEA (RIA, Immunotech, 7.3–57 nmol/L); DHEAS (RIA, Immunotech, 2.4–14.5 µmol/L); insulin-like growth factor (IGF-I) [immune-radiometric analysis (IRMA), Immunotech, 150–450 ng/mL]; GH (ECLIA, Roche, 0.05–10 µg/L); and PRL (ECLIA, Roche, 4.79–23.3 µg/L).

#### Results

Basal hormonal assessment and the results of challenge tests are presented in Table 1. In general, we confirmed central hypothyroidism and hypogonadism, decreased adrenocortical reserve (with basically normal S-cortisol, however insufficient stimulation in insulin test), normal-to-low adrenal androgens, low–normal PRL, and undetectably low IGF-I with no stimulation of GH in either of the stimulation tests: arginin and insulin challenge tests. In Czechia, two confirmation tests are necessary for health insurance approval of GH treatment in adulthood.

**Table 1:** Basic laboratory assessment and results of functional tests.

Hormonal axis/hormone	Basic laboratory assessment	Challenge tests
Thyroid	fT4 3.1 pmol/L; fT3 2.2 pmol/L, TSH 1.3 mIU/L	TSH in TRH test: 1.3–1.7–2.44 mU/L
Gonadal	LH < 0.2 U/L; FSH 0.7 U/L, estradiol < 0.03 nmol/L	LHRH test: FSH 0.2–1.3–0.3 IU/L; LH low–0.2–0.1 IU/L, estradiol 0.4 nmol/L
Adrenocortical	S-cortisol 323 nmol/L, 0.175 nmol/L, ACTH 27.7 ng/L	S-cortisol in insulin test: 453–294–394–273 nmol/L
Adrenal androgens	DHEAS 0.53 µmol/L, DHEA 5.1 nmol/L, androstenedione 2.4 nmol/L	
GH	IGF-1 undetectably low	GH in arginine test: 0.05–0.03–0.04–0.05–0.04 µg/L GH in insulin test: 0.08–0.07–0.05–0.03–0.03 µg/L
Prolactin	6.4 µg/L	

*ACTH, adrenocorticotropin; DHEA, dehydroepiandrosterone; DHEA-S, dehydroepiandrosterone-sulfate; FSH, follicle-stimulating hormone; fT3, free triiodothyronine; fT4, free thyroxin; GH, growth hormone; IGF-1, insulin-like growth factor 1; LH, luteinizing hormone; LHRH, LH-releasing hormone; TRH, thyroliberin; TSH, thyroid stimulating hormone*.

### Other Examinations

**Figure 2:**
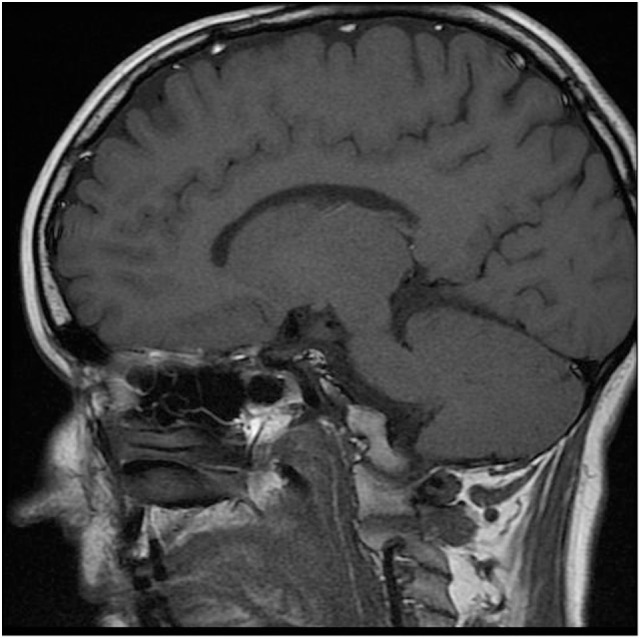
Magnetic resonance imaging of smaller pituitary gland.

The karyotype was normal female (46, XX). Gynecologic examination revealed small ovaries and an infantile uterus. Densitometry showed significantly decreased bone mineral density (BMD) in relation to age (*Z*-score in L-spine −4.5 and in proximal femur −2.1). Molecular biologic examination using sequence analysis confirmed homozygous mutation c.150delA in the PROP1 gene (position of the gene 5q35.3). Unfortunately, complete molecular-genetic analysis including the relatives of our patient was not possible to perform since the whole family lives in a small village in Ukraine. MRI revealed a smaller but otherwise normal pituitary gland (Figure 2).

### Therapy for Central Hypothyroidism and Hypocortisolism

Therapy with levothyroxine was started in May 2013 and continuously titrated up to a final dose of 125 µg daily (reached in September 2016) in order to achieve euthyroid status (fT4 14; fT3 4.2 pmol/L). After confirmation of a decreased adrenocortical reserve and due to decreasing basal S-cortisol to the lower limit of the normal range in repetitive measurements, hydrocortisone 15 mg (10–5–0 mg) was administered daily in September 2013. Calcium 600 mg and vitamin D 400 U daily were started at the same time.

### Auxology and GH Therapy

The titration of GH and growth progression are presented in Table 2. Bone age at the first auxologic examination, when the patient was 26.7 years of age, assessed using the TW3-CARP method (13), was 10.2 years and revealed an extreme delay (−16.5 years!) compared to the patient’s chronologic age. GH therapy was initiated in November 2013 with Humatrope 12, with an initial infantile dose of 1.35 mg/day (0.031 mg/kg/day). During the first months, the dose had to be decreased to 0.3 mg/day (0.007 mg/kg/day) in response to side effects (eyelid edema and ankle pain). After 11 months, the dose was increased to 0.4 mg/day (0.009 mg/kg/day), which was well tolerated. Auxologic examinations including X-rays were performed altogether five times during the treatment with constantly high difference between bone and chronologic ages. The last examination was performed in 2015 at the age of 29.7 years. The difference between bone (TW3-CARP = 12.8 years) and chronologic ages was still high (16.9 years), growth physes were partially open and reached the morphologically perimenarcheal stage, and growth velocity correspondingly decreased to 0.8 cm/year. After induction of menarche, growth velocity increased to 4.5 cm/year and the remaining growth potential contributed to subsequent growth during the next year with a growth velocity of 1.5 cm/year.

**Table 2:** Titration of GH therapy and sex hormone substitution therapy with clinical correlates.

Date	Age (years)	Height (cm/SDS)	GV (cm/year)	GH dose (mg/day)	IGF-1 (ng/ml)	Dose of HST (mg/day)	GUS (mm)	Hormonal cytology (%)
11/13	27.2	149/−2.9		1.35			U 15 × 10	MI 98–2–0 EI 0
12/13	27.3	149/−2.9		0.6	10.0			
2/14	28.3	151.8/−2.46	2.8	0.3	42.0	E 0.1	U 18 × 10	MI 98–2–0 EI 0
5/14	28.5	154.6/−2.01			117.0	E 0.2	U 29 × 10 × 24 O homog.	MI 90–10–0 EI 0
7/14	28.7	158.0/−1.47				E 0.3	U 40 × 9 × 16 O physiol.	MI 80–20–0 EI 0
2–3/15	29.3	160.3/−1.11	8.5	0.4		E 0.5		
5/15	29.5	160.7/−1.04			11.0	E 1	U 40 × 9 × 16 Endom 2 O physiol.	MI 20–80–0 EI 0
9/15	29.8	162.7/−1.04	0.8	0.4		E 2	U 43 × 19 × 28 Endom 3.7	MI 0–99–1 EI 40
11/15	30.0				111.0	E 2 + D 20	U 56 × 15 × 34 Endom 5	MI 0–90–10 EI 5
12/15	30.3					Induction of menarche		
3/16	30.3	162.9/−0.69	4.5	0.4	20.0	Femoston 2/10		
9/16	30.8	163.0/−0.68	0.4	0.4	93.0	Femoston 2/10		
11/16	31.0	163.5/−0.61	1.5	0.4		Femoston 2/10		

*D, dydrogesterone; E, estradiol; EI, eosinophilic index; Endom, endometrium thickness; Femoston 2/10, estradiol 2 mg, followed by estradiol 2 mg + dydrogesterone 10 mg; GH, growth hormone; GUS, gynecologic ultrasonography; GV, growth velocity; IGF-1, insulin-like growth factor 1; HST, hormonal substitution therapy; MI, maturation index; O, ovaries; U, uterus*.

Although the patient’s compliance was not always optimal with suspected multiple missed injections, the patient achieved a final height of 163.5 cm, which exceeded the lower limit of her familial range 158–175 cm (target height by Tanner) (14).

### Sexual Development and Sex Hormone Substitution Treatment (HST)

The patient was examined by a pediatric gynecologist in February 2013 with the following findings: infantile vulva with posterior synechia and per rectum uterus infantilis. The second examination was performed 1 year later (patient related delay) and “mini-estrogenization” with oral estradiol (0.1 mg once daily orally) was started in February 2014. The titration of sex hormone treatment was guided by hormonal cytology—a feasible noninvasive and cheap method routinely used in pediatric gynecology for guidance of hormonal therapy. Normal smear shows the following types of squamous epithelial cells: superficial eosinophillic cells influenced by estrogens, intermediate cyanophillic cells influenced by progesteron, and immature basal and parabasal cells. Eosinophillic index (EI)—the percentage of eosinophillic superficial and cyanophillic parabasal cells—and maturation index (MI)—the percentage study of parabasal, intermediate, and superficial cells/100 cells counted from exfoliated epithelial cells of vaginal smear—describe the maturation of vaginal epithelium. The effect of estrogen is described as MI = 0/10/90, whereas the effect of progesterone is described as MI = 0/90/10 (15, 16).

**Figure 3:**
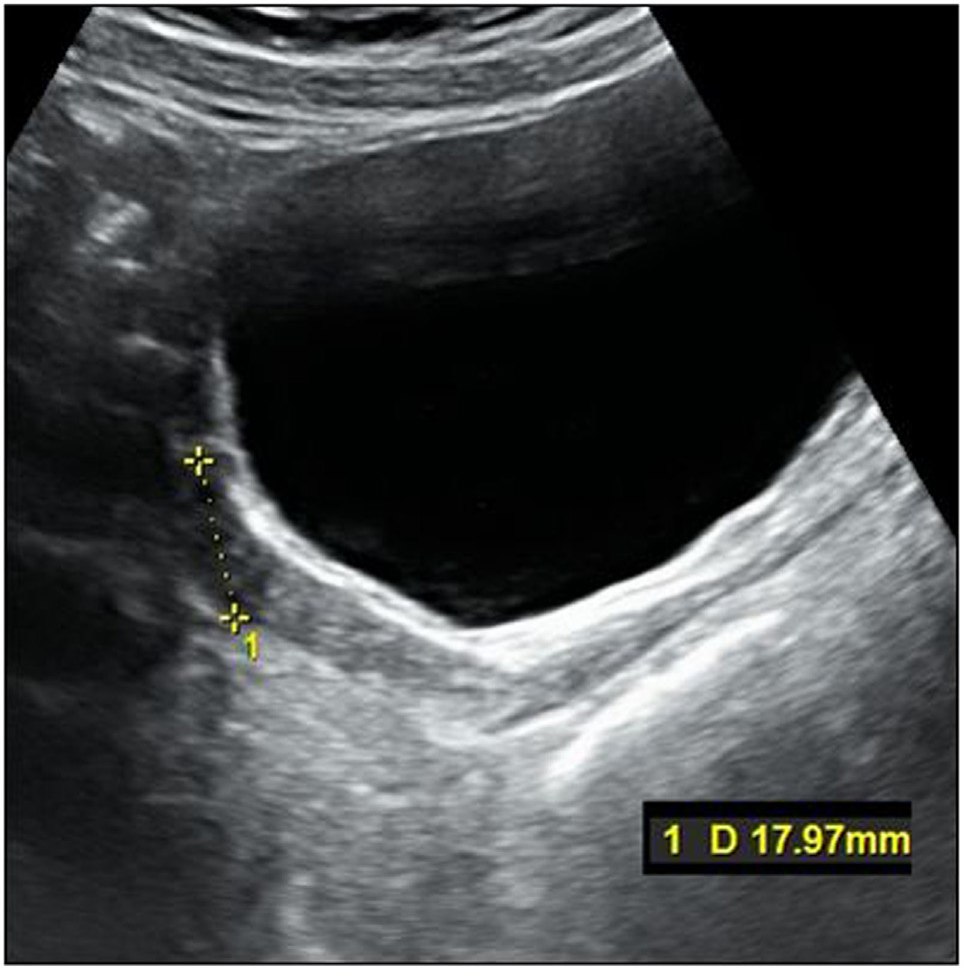
Ultrasonography of uterus at the initiation of the treatment.

The titration of hormonal substitution therapy and findings from hormonal cytology and sonography of the uterus are presented in Table 2 and Figure 3. In general, therapy was well tolerated and a very prompt reaction of estrogen-dependent organs was observed. After 15 months (May 2015), secondary sexual signs developed to B2, PH2, A1, and after 19 months of the treatment (in September 2015) to B4, PH2, A1. In November 2015, the uterus was well developed with a proper endometrium layer. At this point, progesterone (dydrogesterone) 10 mg twice daily for 10 days was added to 2 mg of estradiol; after its withdrawal, menarche was induced on December 11, 2015 and since that time, commercial Femoston 2/10 (estradiol 2 mg daily 5th–25th day of menstrual cycle and dydrogesterone 20 mg 16th–25th day of menstrual cycle) has been administered.

Currently, the patient continues with the established substitution of all above-mentioned pituitary hormones with levothyroxin, hydrocortisone, GH, and sex hormone therapy. The patient reports feeling well and is satisfied with the treatment results.

## Discussion

To our knowledge, such a case of a PROP1 mutation patient with growth retardation and absence of puberty diagnosed very late—at adult age—is quite exceptional. So far, only one case of a hormonally infantile (male) adult patient treated with GH has been presented without therapeutic details (17). Furthermore, two female patients with PROP1 mutation received GH and sex hormone treatment since puberty and successful fertility was achieved later on (18). And finally, PROP1 mutation was identified in an elderly patient with osteoporosis due to previously unrecognized central hypogonadism and GH deficiency and hyperlipidemia due to central hypothyroidism (19). Thus, any comparison with current literature is difficult. However, several interesting points deserve discussion.

The first is the untypically delayed diagnosis due speculative reasons: possible difficult access to specialized healthcare in the Ukrainian countryside and surprising neglect of her continuing childish appearance by her family and by herself, which could not have been explained by psychic deterioration [described elsewhere (20) as a manifestation of PROP1 mutation]. Thus, such a case emphasizes the need for proper pediatric assessment, essential cooperation of the family and early referral of patients with growth retardation and delayed sexual maturation to a specialist.

Secondly, during the decision process about the indication of GH therapy in adulthood, several aspects had to be considered, such as justification of the treatment (physiological and economical) and the dose of GH [the initial pediatric dose (12, 21) had to be reduced to the adult dose very quickly due to adverse side effects]. Our recommendation for “growth hormone naive” cases is to start with lower than recommended doses. It is noteworthy that IGF-1 levels were not useful for monitoring the efficacy of GH treatment since during the follow-up they remained extremely low and their wide fluctuations (and corresponding changes of growth velocity) could have been associated with less than optimal compliance of the patient. Also, due to persistent enormous difference between bone and chronologic ages (possibly explained by delayed reaction of bone to hormonal treatment), the assessment of bone age was not very useful for guidance of GH treatment. Furthermore, psychological aspects were considered regarding the patient’s reaction to sexual maturation.

The third point is connected with the initiation of hydrocortisone treatment despite normal initial cortisol levels and possible deleterious effects on growth and bone metabolism. Our decision was guided by decreased adrenal reserve in a challenge test, the trend to a decrease in basal cortisol in repetitive measurements and rapid up-titration of thyroxin, which could have increased the demands on the adrenocortical axis, and furthermore, such doses as ours were safe regarding bone metabolism (22) and growth (23). On the other hand, hydrocortison substitution does not seem to require dose adjustment after initiation of GH replacement (24).

Finally, the fourth point to be discussed is sex hormone substitution therapy. Our estradiol starting dose was not observed to effect growth, although a biphasic effect of estrogen on growth was described, with high concentrations leading to growth cessation, while low concentrations to its stimulation (25). On the other hand, “mini-estrogenization” had significant effects on previously hormone-naive, estrogen-dependent tissues (uterus, breast). In our patient, contrary to some observations (25), increased GH doses (due to HST) were not required. As typically seen in PROP1 mutation patients, our patient also failed to develop axillary and pubic hair (26). In addition, low values of dehydroepiandrosterone sulfate (DHEA-S) have been reported in the presence of a normal pituitary-adrenal axis in PROP1 mutation patients (27).

## Conclusion

We present a case of an adult female patient with PROP1 mutation, which is unique particularly due to surprisingly late diagnosis of CPHD which included central hypogonadism with primary amenorrhea and growth retardation. Our case emphasizes the need for early diagnosis of CPHD based on proper pediatric assessment, good cooperation with the family, and early referral to an endocrinologist in patients with these symptoms. On the other hand, even an adult patient can profit from very cautiously combined GH therapy and sex hormone therapy, and can achieve normal height and regular menstrual cycle.

## Ethics Statement

The case report was written with the recommendations of the Declaration of Helsinki. The patient is described anonymously and gave written informed consent with the publication. The presentation of the case report was approved by Ethics Committee of 3rd Faculty of Medicine, Charles University, Prague.

## Author Contributions

LB diagnosed the patient and wrote the manuscript. IC and BK led the growth hormone treatment and contributed to the preparation of the manuscript (GH treatment part and discussion part). JS and RP led the hormonal substitution treatment and contributed to the writing of the manuscript (HST part and discussion part). PS did all the auxology and contributed to the writing of the manuscript (auxology part and discussion part).

## Conflict of Interest Statement

The authors declare that the research was conducted in the absence of any commercial or financial relationships that could be construed as a potential conflict of interest.
